# Cesarean section at full dilatation in the first birth is not associated with an increased risk of subsequent miscarriage: A historical cohort study

**DOI:** 10.1111/aogs.14936

**Published:** 2024-08-04

**Authors:** Andrea Woolner, Edwin Amalraj Raja, Mairead Black

**Affiliations:** ^1^ Aberdeen Center for Women's Health Research (ACWHR), Institute Applied Health Sciences, School of Medicine, Medical Sciences and Nutrition University of Aberdeen Aberdeen UK; ^2^ Medical statistics, Institute Applied Health Sciences, School of Medicine, Medical Sciences and Nutrition University of Aberdeen Aberdeen UK

**Keywords:** cesarean section at full dilatation, miscarriage, second stage cesarean section

## Abstract

**Introduction:**

Cesarean section at full dilatation has been associated with an increased risk of subsequent preterm birth. We hypothesized that there may be an increased risk of miscarriage in pregnancies that follow cesarean section at full dilatation. This study aimed to determine if a first‐term (≥37 weeks) cesarean section at full dilatation is associated with an increased risk of miscarriage in the next pregnancy.

**Material and Methods:**

A historical cohort study was conducted using routinely collected hospital data within the Aberdeen Maternity and Neonatal Databank (AMND). The population included were women who had a first‐term birth and who had a second birth recorded within the AMND. Logistic and multinomial regression was used to determine any association with miscarriage at any gestation and for early (<13 weeks gestation) and late (13–23 + 6 weeks gestation) miscarriage, with cesarean section at full dilatation defined as the exposure. Miscarriage in the second pregnancy (spontaneous loss of intrauterine pregnancy prior to 24 weeks gestation) was the primary outcome.

**Results:**

In total, 33 452 women were included. Women who had a first cesarean section at full dilatation were no more likely to have a miscarriage at any gestation than women with all other modes of first birth (including all vaginal births, planned CS, and the first stage of labor (<10 cm dilated CS)) [adjusted OR 0.84 (0.66–1.08); *p* = 0.18]. There was no association with early or late miscarriage after a CSfd, though the sample size for late miscarriage was small.

**Conclusions:**

This is the first observational study to investigate the risk of miscarriage following first‐term CSfd. We found no association between miscarriage at any gestation following a first‐term CSfd compared to all other modes of first birth.

## Graphical abstract

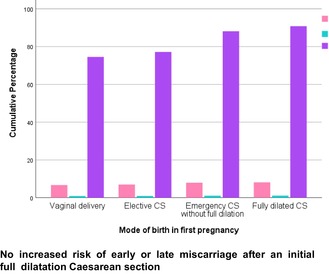

**Abbreviations**
AMND: Aberdeen Maternity and Neonatal Databank
BMI: body mass index
CS: cesarean section
CSfd: cesarean section at full cervical dilation
sPTB: spontaneous preterm birth

**Key message**

In this historical cohort study, we found no association with miscarriage (early or late) and a history of having a cesarean section at full dilatation in the first birth. Large population‐based studies are needed to confirm or refute our findings.

## 1 INTRODUCTION

When cesarean section (CS) is undertaken at full cervical dilation (CSfd), there is clear epidemiological evidence of an increased risk of spontaneous preterm birth (sPTB) in subsequent pregnancies.^1^, ^2^, ^3^, ^4^, ^5^, ^6^, ^7^ The biological cause is unknown.^7^ Theories, such as injury to the lower uterine segment or cervix causing cervical weakness; adhesions or scarring from CS surgery which detrimentally affects uteroplacental implantation or blood flow of the next pregnancy, have been proposed.^8^, ^9^, ^10^, ^11^ However, the risk of miscarriage after CSfd is much less studied. It is plausible that late miscarriage has a similar underlying pathophysiological process to preterm labor related to cervical incompetence or uteroplacental implantation. We hypothesized that CSfd could detrimentally affect the risk of early miscarriage through changes to uteroplacental implantation, blood flow, or cervical integrity. In addition, with growing evidence of the impact CS has on menstrual problems^12^ and fertility,^13^ we hypothesized that niche formation and scarring could also affect the risk of miscarriage. Through changes in implantation or changing the structure of the endometrium within the uterus, we hypothesized that this may be of particular relevance for CSfd where incisions are made in the overstretched LUS and subsequent risk of early or late miscarriage.

There is a lack of research on the risk of miscarriage after a prior CSfd. Zhao et al.^14^ suggest having any type of CS increased the risk of miscarriage during subsequent assisted reproduction but did not specifically investigate the impact of having a CSfd. Vyas et al.^15^ conducted a case–control study and suggested that cervical insufficiency was increased after a prolonged second stage of labor in a previous birth but did not specifically investigate prior CSfd. Another study investigated the risk of recurrent late miscarriage or sPTB in women who have had a CSfd followed by a first sPTB or late miscarriage.^16^ Watson et al.^16^ suggest that recurrent late miscarriage and sPTB are greater in women with prior CSfd and a prior sPTB or late miscarriage, though the sample size was small. Interventions such as transabdominal cerclage are suggested to reduce the risk of recurrent late miscarriage or preterm birth after CSfd by the same researchers,^17^ however, the risk of early or late miscarriage in the pregnancy immediately after CSfd remains unknown.

We present the first observational study investigating the risk of miscarriage at any gestation in pregnancies after a CSfd in the first‐term birth using a high‐quality and validated population‐based data source in Aberdeen, UK. The Aberdeen Maternity and Neonatal Databank (AMND) provides a rare opportunity to study subsequent pregnancies in a stable population with detailed availability of confounding factors and is one of few sources with miscarriage data.^18^

## 2 MATERIAL AND METHODS

A population‐based historical cohort study was conducted using routinely collected hospital data to compare the risk of miscarriage in the second pregnancy for women with and without a history of a CSfd in the first term birth. The population included all women who had a term livebirth (defined as a baby born at ≥37 weeks gestation) in their first pregnancy and who had a miscarriage <24 weeks or birth ≥24 weeks gestation in their second pregnancy. Women were included where their first and second pregnancies were recorded within the AMND. First pregnancies resulting in a term birth by any mode of birth were included from 1976 until 2016. Second pregnancies were included from 1977 to 2017. The exposed cohort was defined as all women who had a CSfd in their first pregnancy. The unexposed cohort included all women who did not have a CSfd in their first pregnancy (including all vaginal births (spontaneous or assisted vaginal birth), elective CS (women not in labor at the time of CS), and women who had a CS in the first stage of labor (at <10 cm dilated)). The binary primary outcome was miscarriage, defined as a spontaneous loss of an intrauterine pregnancy at gestational <24 weeks. The data only included women who had a miscarriage recorded within the hospital. We also stratified according to gestation at the time of miscarriage to early (<13 weeks) and late miscarriage (13–23 + 6 weeks). A further analysis investigated very late miscarriage (17–23 + 6 weeks). Women with multiple pregnancies in their first or second births were excluded. Women with any preterm delivery in the first pregnancy (<37 weeks gestation); and where the first pregnancy ended in miscarriage, ectopic pregnancy, termination of pregnancy, or molar pregnancy were excluded. Women whose second births resulted in an ectopic pregnancy, molar pregnancy, or termination of pregnancy were excluded.

Second pregnancy outcomes were compared between the exposed (women where first birth was a CSfd) and unexposed group (all other modes of first birth). Subgroup analyses were performed comparing women with prior CSfd to each individual prior mode of birth including any vaginal birth (inclusive of spontaneous vaginal birth, non‐rotational forceps, rotational forceps, vacuum as well as breech vaginal births), elective CS, first stage CS (CS prior to full dilatation in the first stage of labor where the cervix was ≤9 cm dilated). First‐stage CS was identified within the AMND data by selecting women with a first stage of labor recorded, confirming they had been in labor but with no second stage documented assuming CS was performed prior to 10 cm dilated. CSfd was defined by identifying women with a CS where a first and second stage of labor was recorded but the eventual mode of birth was a CS.

This study is reported in accordance with STROBE guidance.^19^ Approval was obtained from the AMND steering committee (reference: AMND2020‐01) to undertake this research. The AMND steering committee has overarching ethical approval for studies that use pseudo‐anonymized data with no data linkage and therefore formal ethics approval was not required. The AMND holds routinely collected pregnancy data for all women who gave birth in Aberdeen Maternity Hospital from 1949 until present 2017.^20^ All pregnancy records were included automatically in the AMND until 2017,^20^ and the information was entered routinely for all women under the jurisdiction of Aberdeen Maternity Hospital until 2017, which is the only maternity hospital in the area. Prior to 1976, CSfd was not recorded within the AMND therefore women were included only after this date. A pseudo‐anonymized dataset was provided to the researchers and was analyzed within the Grampian data safe haven in accordance with data protection laws. No data linkage was performed, and no raw data was transferred out of the safe haven. Data can be made available by applying to the AMND for permission (amnd@abdn.ac.uk). This study was carried out in conjunction with a published study investigating the risk of sPTB after CSfd.^1^

### 2.1 Definitions of outcomes

Gestation at miscarriage or birth is coded within the AMND according to the due date that was estimated by the first‐trimester ultrasound scan when available from hospital records (from 1986 onwards) and otherwise by the last menstrual period date that was recorded at the first antenatal booking and the date of birth.

### 2.2 Definition of confounders

Socioeconomic status was recorded using the Scottish index of multiple deprivation (SIMD 2016),^21^ where 1 is the least deprived and 10 is the most deprived and this was recorded routinely in the AMND. SIMD^21^ is a marker of socioeconomic status for zip code geographical areas in Scotland, and is an objective measure of how deprived that area is, and uses information from seven categories including income, employment, education, and health, access to services, crime, and housing. Maternal age at baby's birth was collected routinely by the AMND from the hospital medical records. Smoking status was self‐reported at the time of antenatal booking and documented within the hospital record from which it was collected for inclusion within the AMND. Body mass index (BMI) is calculated from the booking height and weight measurements undertaken at the antenatal booking appointment.

### 2.3 Statistical analyses

All data were stored and analyzed using SPSS software (IBM Corp. Released 2018. IBM SPSS Statistics for Windows, Version 25.0, IBM Corp, Armonk, NY.). Parametric and non‐parametric tests were used to compare normally and not normally distributed variables, respectively. Chi‐square was used to compare categorical variables. Binary logistic regression models were used to look for any associations between the exposure (history of CSfd in the first pregnancy) and the primary outcome (miscarriage at any gestation <24 weeks). Multinominal logistic regression was used to determine any association between the exposure (history of CSfd in the first pregnancy) and categorical outcomes early miscarriage (<13 weeks gestation) or late miscarriage (13–23 + 6 weeks gestation; then, in an analysis, miscarriage up to 16 + 6 weeks and miscarriage from 17 to 23 + 6 weeks gestation) with births at >24 weeks used as the reference category. Multivariable models were used to adjust for potential confounding factors. The strength of the measure of association was estimated using odds ratios (ORs), adjusted OR (aOR), and 95% confidence intervals (95% CI). Where the 95% CI does not include 1 the OR was considered statistically significant. P‐values of less than 0.05 were deemed statistically significant.

Potential confounding factors were included from the first pregnancy including maternal age, smoking status, socioeconomic status, and year. The year of delivery was included to account for potential confounding due to changes in practice over time. There was substantial missing data in the second pregnancy where women miscarried compared to women who had a second pregnancy beyond 24 weeks gestation, therefore the data was deemed not missing at random, and so all potential confounders were included from the first pregnancy only, for all women. Complete case analyses were conducted. Where missing data was >5% for covariates, a sensitivity analysis was carried out for adjusted analyses excluding smoking, BMI, and socioeconomic status individually from the multivariate analyses. Multiple imputation was used to impute values for smoking, BMI, and socioeconomic status where missing values exceeded 5% and separate multivariate analyses were performed with imputed values using the automatic method within SPSS software (“the Automatic method scans the data and uses the monotone method if the data show a monotone pattern of missing values; otherwise, fully conditional specification is used. The fully conditional specification (FCS) imputation method imputes values in the order specified in the Analysis Variables list”)^24^.

## 3 RESULTS

A cohort of 33 452 women were included who had a first and second pregnancy recorded within AMND and where the first pregnancy resulted in a term live birth. In this study, 27 452 women had a vaginal birth in the first pregnancy, 970 had a planned non‐emergency CS, 4051 had a CS performed in the first stage of labor and 979 had a CS performed in the second stage of labor. First pregnancy and birth characteristics are shown in Table 1.

**TABLE 1:** First pregnancy demographic and obstetric characteristics (1976–2016).

Index pregnancy variable	Mode of pregnancy in index pregnancy				
*n* (%)	Vaginal birth, *N* = 27 452	Elective CS, *N* = 970	First stage CS, *N* = 4051	Second stage CS, *N* = 979	*p*‐value
Age					
16–25	14 840 (54.1)	330 (34.0)	1557 (38.4)	384 (39.2)	<0.01
26–35	12 157 (44.3)	590 (60.8)	2310 (57.0)	561 (57.3)	
>35	454 (1.7)	50 (5.2)	184 (4.5)	34 (3.5)	
Mean (SD)	25.4 (4.9)	27.2 (5.2)	26.9 (5.1)	26.7 (4.9)	
Missing	1	0	0	0	
Smoking					
Non smoker	14 100 (51.4)	549 (56.6)	2539 (62.7)	643 (65.7)	<0.01
Smoker	6879 (25.1)	178 (18.4)	757 (18.7)	137 (14.0)	
Ex smoker	1531 (5.6)	50 (5.2)	282 (7.0)	76 (7.8)	
Missing	4942 (18.0)	193 (19.9)	473 (11.7)	123 (12.5)	
Body mass index					
<20	2553 (9.3)	76 (7.8)	213 (5.3)	50 (5.1)	<0.01
20–25	13 273 (48.3)	428 (44.1)	1672 (41.2)	418 (42.7)	
25–30	5634 (20.5)	238 (24.5)	1088 (26.9)	274 (28.0)	
>30	2038 (7.4)	105 (10.8)	675 (16.7)	137 (1.3)	
Missing	3954 (14.4)	123 (12.7)	403 (9.9)	100 (10.2)	
Deprivation (SIMD 2016)					
1–5 (least deprived)	7928 (28.9)	240 (24.7)	1067 (26.3)	230 (23.5)	
6–10 (most deprived)	18 276 (66.6)	661 (68.1)	2714 (67.0)	680 (69.5)	<0.01
Missing	1248 (4.5)	69 (7.1)	270 (6.7)	69 (7.0)	
Diabetes (any)					
Yes	258 (0.9)	39 (4.0)	106 (2.6)	21 (2.1)	<0.01
No	27 194 (99.1)	931 (96.0)	3945 (97.4)	958 (97.9)	
Antepartum hemorrhage					
Yes	2454 (8.9)	109 (11.2)	439 (10.8)	87 (8.9)	<0.01
No	24 998 (91.1)	861 (88.8)	3612 (89.2)	892 (91.1)	
Pre‐eclampsia					
Yes	7692 (28.0)	248 (25.6)	1429 (35.3)	351 (35.9)	<0.01
No	19 760 (72.0)	722 (74.4)	2622 (64.7)	628 (64.1)	
Threatened miscarriage					
Yes	4646 (16.9)	199 (20.5)	665 (16.4)	159 (16.2)	0.02
No	22 806 (83.1)	771 (79.5)	3386 (83.6)	820 (83.8)	
Labor type					
Spontaneous	19 128 (69.7)	n/a	1937 (47.8)	370 (37.8)	<0.01
Induced	8322 (30.3)		2114 (52.2)	609 (62.2)	
Missing	2		0	0	
Length first stage labor (hrs)					<0.01
Median (IQR)	8 (7)	n/a	n/a	11 (7)	
Missing	5399 (19.7)			158 (16.1)	
Length second stage labor (hrs)					<0.01
Median (IQR)	1 (1)	n/a	n/a	3 (2)	
Missing	1774 (6.5)			109 (11.1)	
Neonatal admission					
Yes	1588 (5.8)	110 (11.3)	533 (13.2)	151 (15.4)	<0.01
No	25 864 (94.2)	860 (88.7)	3518 (86.8)	828 (84.6)	
Blood loss					<0.01
Median (IQR)	200 (150)	450 (300)	500 (320)	500 (400)	
Missing	77 (0.3)	16 (1.6)	32 (0.8)	4 (0.4)	
Presentation fetal head					
OA	24 220 (88.2)	n/a^a^	2552 (63.0)	361 (36.9)	<0.01
OP	1511 (5.5)		643 (15.9)	282 (28.8)	
OT	1365 (5.0)		343 (8.5)	220 (22.5)	
Breech	230 (0.8)		423 (10.4)	93 (9.5)	
Other	126 (3.3)		77 (1.8)	22 (2.2)	
Gestation at birth (weeks)					
Median (IQR)	40 (2)	39 (1)	40 (2)	40 (2)	<0.01
Birthweight (g)					
Mean (SD)	3360.8 (451.1)	3542.7 (552.7)	3418.3 (522.4)	3500.3 (460.0)	<0.01
Missing	0	0	1	0	
Decade of birth					
1976–1985	7903 (28.8)	202 (20.8)	673	161	<0.01
1986–1995	9063 (33.0)	280 (28.9)	977	280	
1996–2005	5870 (21.4)	232 (23.9)	1353	308	
2006–2016	4615 (16.8)	256 (26.4)	1048	230	

Abbreviations: CS, cesarean section, IQR, interquartile range; OA, occiput anterior; OP, occiput posterior; OT, occiput transverse; SIMD, Scottish Index of Multiple Deprivation.

^a^ Excluded due to disclosure risks and as not clinically relevant information to include elective CS in this analysis.

**FIGURE 1:**
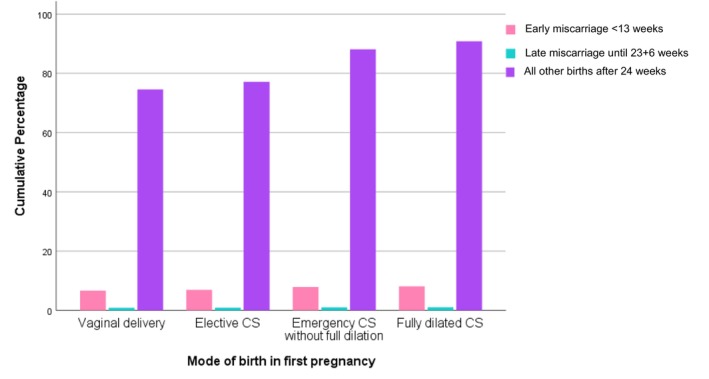
Bar chart of second pregnancy outcomes according to mode of birth in first pregnancy.

Second pregnancies were included from 1977 to 2017. Of all second pregnancies, 3191 women had a miscarriage in their second pregnancy and 30 261 women had a birth from 24 weeks gestation including 140 women who had a stillbirth. Gestation at miscarriage was recorded for 3057 women; 2699 (88.3%) had a miscarriage in the first trimester (up to 12 + 6 weeks of gestation) and 358 (11.7%) had a miscarriage between 13 and 23 + 6 weeks. Figure 1 shows the percentage of women who had an early or late miscarriage or birth from 24 weeks gestation according to the mode of first birth. Table 2 shows the results of comparative analyses according to the first mode of birth for the primary outcome of miscarriage (at any gestation), as well as the results for the secondary outcomes according to gestation at miscarriage (early or late miscarriage) compared to all other births >24 weeks gestation. There was no significant difference in the proportion of women who miscarried at any gestation in their second pregnancy (Table 2). 8.1% (79/979) of women who had a CSfd in their first pregnancy had a miscarriage at <24 weeks gestation; compared to 9.5% (3112/32 471) of women who had any other mode of birth in their first birth. Early (up to 12 + 6 weeks) miscarriage for women who had a CSfd in the first pregnancy occurred in 6.7% (65/977) of second pregnancies and was 8.1% (2634/32 341) for women with any other mode of first birth. Late miscarriage (13 weeks to 23 + 6 weeks) occurred in 1.2% (12/977) of second pregnancies for women with a first CSfd, compared to 1.1% (346/32 341) of second pregnancies for women with any other mode of first birth. Similarly, no difference was found for the risk of miscarriage in the second birth when subgroup analyses were performed for an individual mode of first birth vs CSfd (vaginal births only vs. CSfd; elective CS vs. CSfd; first stage CS vs. CSfd). Multiple imputation was employed for smoking, socioeconomic status and BMI and this confirmed no association between mode of first birth and outcome of miscarriage in the second pregnancy (all other modes of first birth vs. CSfd: OR 0.90 [95% CI 0.70–1.15]; vaginal births only vs. CSfd in the first birth: OR 0.91 [95% CI 0.71–1.17]). Tables 3, 4, 5 demonstrate comparison for the outcome of miscarriage between second stage CS compared to vaginal births only (Table 3), first stage CS only (Table 4) and elective CS only (Table 5) and demonstrated no increased association with miscarriage in the second pregnancy.

**TABLE 2:** Outcome of second pregnancies according to mode of birth in the first pregnancy (*N* = 33 452) and comparison according to first mode of birth.

Second pregnancy outcome, *n*(%)	Mode of birth in first pregnancy				Comparison of all births vs second stage CS in first pregnancy		
	Vaginal birth	Elective CS	First stage CS	Second stage CS	Unadjusted OR (95% CI)	Adjusted OR (95% CI)	*p*‐value
Miscarriage (up to 23 + 6 weeks) (*n* = 33 452)^a^	*N* = 27 452	*N* = 970	*N* = 4051	*n* = 979	0.82 (0.64–1.05)	1.08 (0.15–7.98)	0.94
Yes	2617 (9.5)	102 (10.5)	393 (9.7)	79 (8.1)			
No	24 835 (90.5)	868 (89.5)	3658 (90.3)	900 (91.9)			
Miscarriage (early or late) (*N* = 33 318)	*N* = 27 348^c^	*N* = 963^c^	*N* = 4030^c^	*N* = 977^c^			
First‐trimester miscarriage^b^	2224 (8.1)	87 (9.0)	323 (8.0)	65 (6.7)	1.24 (0.96–1.60)	1.26 (0.88–1.81)	0.21
Second‐trimester miscarriage^b^	289 (1.1)	8 (0.8)	49 (1.2)	12 (1.2)	0.88 (0.50–1.58)	0.64 (0.21–2.02)	0.45
All other births	24 835 (90.1)	868 (89.5)	3658 (90.8)	900 (92.1)	1.00	1.00	

Abbreviation: CS, cesarean section.

a Adjusted for maternal age at first birth, deprivation (P1), smoking (P1), interpregnancy interval, BMI (P1), and year of delivery.

b Adjusted for maternal age at first birth, deprivation (P1), smoking (P1), BMI (P1), and year of delivery.

^c^ Total sample numbers are given for subgroup analysis where small number of miscarriages the gestation at miscarriage was missing.

**TABLE 3:** Comparison of miscarriage according to mode of birth in the first pregnancy (second stage CS vs. vaginal births only *N* = 28 431).

Second pregnancy outcome	Comparison vaginal births only vs second stage CS in the first pregnancy		
	Unadjusted OR (95% CI)	Adjusted OR (95% CI)	*p*‐value
All miscarriages (up to 24 weeks)^a^	0.83 (0.66–1.05)	1.05 (0.14–7.90)	0.96
First‐trimester miscarriage (up to 12th week)^b^	1.24 (0.96–1.60)	1.14 (0.87–1.50)	0.33
Second‐trimester miscarriage (13–23 weeks)^b^	0.87 (0.49–1.56)	0.79 (0.38–1.61)	0.51

^a^ Adjusted for maternal age at first birth, deprivation (P1), smoking (P1), interpregnancy interval, BMI (P1), and year of delivery.

^b^ Adjusted for maternal age at first birth, deprivation (P1), smoking (P1), BMI (P1), and year of delivery.

**TABLE 4:** Comparison of miscarriage according to the mode of birth in first pregnancy (second stage CS vs. elective CS, *N* = 1949).

Second pregnancy outcome	Comparison vaginal births only vs second stage CS in the first pregnancy		
	Unadjusted OR (95% CI)	Adjusted OR (95% CI)	*p*‐value
All miscarriages (up to 24 weeks)^a^	0.75 (0.55–1.02)	1.37 (0.14–13.45)	0.79
First‐trimester miscarriage (up to 12th week)^b^	1.39 (0.99–1.94)	1.26 (0.88–1.81)	0.21
Second‐trimester miscarriage (13–23 weeks)^b^	0.69 (0.28–1.70)	0.64 (0.21–2.02)	0.45

^a^ Adjusted for maternal age at first birth, deprivation (P1), smoking (P1), interpregnancy interval, BMI (P1), and year of delivery.

^b^ Adjusted for maternal age at first birth, deprivation (P1), smoking (P1), BMI (P1), and year of delivery.

**TABLE 5:** Comparison of miscarriage according to the mode of birth in the first pregnancy (second stage CS vs. first stage CS, *N* = 5030).

Second pregnancy outcome	Comparison vaginal births only vs second stage CS in the first pregnancy		
	Unadjusted OR (95% CI)	Adjusted OR (95% CI)	*p*‐value
All miscarriages (up to 24 weeks)^a^	0.82 (0.64–1.05)	0.74 (0.04–14.64)	0.85
First‐trimester miscarriage (up to 12th week)^b^	1.22 (0.93–1.61)	1.14 (0.85–1.53)	0.38
Second‐trimester miscarriage (13–23 weeks)^b^	1.01 (0.53–1.90)	1.10 (0.51–2.38)	0.81

^a^ Adjusted for maternal age at first birth, deprivation (P1), smoking (P1), interpregnancy interval, BMI (P1), and year of delivery.

^b^ Adjusted for maternal age at first birth, deprivation (P1), smoking (P1), BMI (P1), and year of delivery.

A secondary outcome of very late miscarriage in the second birth was investigated (second births from 24 weeks gestation considered as the reference category) and found no significant difference when comparing first pregnancy mode of birth with CSfd compared to all other first births. The percentage of women who had miscarriage from 17 to 23 + 6 weeks was <0.5% for all women regardless of mode of first birth with no significant difference seen in women who had a CSfd in their first birth compared to all other modes of birth for miscarriage up to 16 + 6 weeks (unadjusted OR (uOR) 1.22 [95% CI 0.96–1.55], adjusted OR (aOR) 1.21 [95% CI 0.87–1.45] *p* = 0.39) or miscarriage from 17 to 23 + 6 weeks gestation (uOR) 0.80 [95% CI 0.25–2.53], (aOR) 0.67 ([95% CI 0.16 to 1.80] *p* = 0.59). Though the total sample where miscarriage occurred from 17 weeks to 23 + 6 weeks is very small (*n* = 81) for all included women.

## 4 DISCUSSION

Women with a prior CSfd do not appear to be at increased risk of miscarriage in the subsequent pregnancy. This is the first observational study to investigate the risk of miscarriage at any gestation in the second birth after a first term CSfd.

To our knowledge this is the first observational study which has investigated the risk of miscarriage following a CSfd in the first term birth. We adjusted for year of birth in the multivariate analysis to minimize confounding caused by change in practice over time. As described in our study investigating sPTB after CSfd,^1^ one of the strengths of this study is the use of the AMND as the data source.^18^ The AMND is a validated and high quality data source of routinely collected hospital data, having been used for multiple large observational studies with access to extensive co‐variate data.^20^ The outmigration rate from the Aberdeen area is low (3.8%) meaning most women remain in Aberdeen for their pregnancies,^20^ therefore this is an ideal data source to study subsequent pregnancy outcomes. Whilst there remains a risk that some women will not have had their first and second pregnancies recorded within the same hospital the risk in this population is known to be low. We believe this is particularly important in this research as the AMND is one of the few databases where all miscarriages involving hospital attendance, at any gestation, are recorded.

However, this study has limitations. The small sample size for late miscarriage means there is a risk of type 2 error and thus, our findings on late miscarriage should be interpreted with caution. By including only subsequent pregnancies after a first birth, the results may not be generalizable to multiparous women. In addition, it is not possible to know how many women did not plan a pregnancy after an initial birth. Conducting a study on subsequent pregnancy outcomes means it is possible that a proportion of second pregnancies will not be captured. This is of particular concern for the most recent first pregnancies where a second pregnancy may not have occurred yet. In addition, because not all women having a miscarriage will seek hospital treatment there is a risk that some miscarriages would have been missed from the database. This is particularly relevant over time, as historically women were less likely to seek hospital care for early miscarriages. Similarly, biochemical pregnancies were not collected routinely in this database and thus, we cannot tell if there is any difference in biochemical pregnancies after CSfd. Unfortunately, the data on demographic information and year of miscarriage was incomplete for the second pregnancies where the second pregnancy ended in miscarriage. Therefore, we included potential confounders in the multivariate analyses from the first pregnancy, but we acknowledge this a limitation. Whilst we recognize the limitation this poses, particularly for maternal age which is a significant risk factor for miscarriage, we felt that the age at first pregnancy was an acceptable surrogate. As previously reported,^1^ the method by which CSfd was recorded within the AMND is a potential limitation, as it was defined according to the documentation of a date and time of second stage of labor onset. We did not include indication for CS in the analysis and this is a limitation in our study. Other risk factors for miscarriage such as antiphospholipid syndrome, polycystic ovarian syndrome (PCOS), gestational diabetes or incidence of concurrent infection were not available to researchers thus present a limitation of the findings. We did not have access to CS niche measurements as this was not historically collected, and we did not have information on CS scar pregnancies.

Quinones et al.^22^ and Vyas et al.^15^ hypothesized previously that the duration of second stage of labor and CSfd could lead to a subsequent risk of PTB or cervical insufficiency respectively. However, in our analyses there was no association with any of the stratifications of miscarriage and a history of CSfd, where notably women were also more likely to have a prolonged second stage compared to women who had a first vaginal birth (Table 1). Our findings do not align with previous reports of a clear association between CSfd and subsequent sPTB,^1^, ^2^, ^3^, ^4^, ^5^, ^6^, ^7^ including our previous study^1^ using the same population database. Our findings suggest that any detrimental effects of having a CSfd do not appear to affect pregnancy outcomes in the first and early second trimester (up to <24 weeks gestation). In our previous study^1^ investigating sPTB after a CSfd we noted that the prevalence of sPTB appeared to be greater after 34 weeks, suggesting that any effect of having a CSfd increased the chance of having a late preterm birth. However, Wood et al.^5^ found a more significant number of preterm births <32 weeks in their sample of Canadian data. Notably Wood et al.^5^ included 20–24 weeks gestation within their definition of preterm birth. The global differences in definition of miscarriage make meaningful comparisons between studies on miscarriage, stillbirth and preterm birth challenging. Nonetheless we acknowledge our sample had a small number of very late miscarriages and evidence remains limited on the risk of very late miscarriage after CSfd.

In contrast, research has suggested that CS does affect subsequent pregnancy outcome in couples using assisted reproduction.^13^, ^14^ Gale et al.^13^ found that after a CS in labor (but not specifically CSfd) that livebirth rates were lower following assisted reproduction compared to women with a first vaginal birth. Though notably there was no increased risk of miscarriage according to mode of first birth in their study.^13^ A similar study of couples undergoing assisted reproduction agrees, suggesting that livebirth, clinical pregnancy rates and implantation rates were lower in women with a prior CS (not specifically a CSfd) but miscarriage rates were unaffected.^23^ Findings indicate that having a CS scar may affect fertility in couples seeking assisted reproduction, although the biological rationale for such an association is not clear. In addition, it is not known if CSfd affects the subsequent risk of secondary infertility. Growing evidence suggests that abnormal uterine bleeding is associated with CS scar niche presence,^12^ thus it remains plausible that CS and CSfd could affect reproductive outcomes.

A systematic review of observational studies^20^ highlighted that the evidence for the risk of subsequent miscarriage after any type of CS (not specifically CSfd) was inconsistent over time. A study using births until 1997 using the same database as our research (AMND) which differentiated between elective and emergency CS (but did not study CSfd specifically) found that any type of CS did not increase the chance of subsequent miscarriage,^19^ though an earlier study^21^ of births by any CS from 1964 until 1983 in Aberdeen showed an increased risk of miscarriage after any CS which may suggest a historical influence possibly due to surgical techniques. Notably, in the systematic review,^20^ none of the included studies adjusted for confounding factors and the majority of studies showed no association between any prior CS and miscarriage in the next pregnancy in keeping with our findings.

Many of the causes of first‐ and second‐trimester miscarriage are unknown. Whilst we found no association in our population, further observational research to investigate fertility and risk of early and late miscarriage after CSfd is needed. Further research to understand the impact of the sonographic appearance of CS scar niches in future pregnancies on subsequent reproduction is needed.

## 5 CONCLUSION

Cesarean section at full dilatation in the first pregnancy was not associated with an increased risk of miscarriage in the next pregnancy in this study. Our findings are reassuring to women and healthcare professionals. Whilst no association was seen with late miscarriage, we recommend caution as the sample size for very late miscarriage was small in this study. Others have raised the possibility of increased risk of recurrent sPTB or late miscarriage^16^ after CSfd, and thus existing surveillance in this group should continue as per local clinical practice guidelines given the lack of evidence until further larger studies can confirm or refute our findings.

## AUTHOR CONTRIBUTIONS

Andrea Woolner and Mairead Black devised the concept for the study. All authors contributed to the study design. Andrea Woolner and Edwin Amalraj Raja conducted the data analyses. Andrea Woolner wrote the first and subsequent drafts of the manuscript; all authors edited each and the final draft of the manuscript.

## FUNDING INFORMATION

This study was funded by the Glasgow Children's Hospital Charity (GCHC).

## CONFLICT OF INTEREST STATEMENT

Andrea Woolner received travel expenses only from System C (owner of Badgernet Maternity electronic medical records systems) and travel expenses from the Scottish Government to speak at their national miscarriage event. Edwin Amalraj Raja and Mairead Black have no conflicts of interest to report.

## ETHICS STATEMENT

Approval was obtained from the AMND steering committee (reference: AMND2020‐01) to undertake this research on January 22, 2020. The AMND steering committee has overarching ethical approval for studies which use pseudo‐anonymized data with no data linkage and therefore formal ethics approval was not required.
